# Correction to ‘Homogenized boundary conditions and resonance effects in Faraday cages’

**DOI:** 10.1098/rspa.2017.0331

**Published:** 2017-06-14

**Authors:** D. P. Hewett, I. J. Hewitt

**Affiliations:** Mathematical Institute, University of Oxford, Oxford, UK

*Proc. R. Soc. A* **472**, 20160062 (Published online May 4 2016) (doi:10.1098/rspa.2016.0062)

— The Neumann boundary conditions (3.6), (3.9), (3.26), (A 11), (A 15), (A 22), (A 27) and (A 31) are applicable only when *K* is symmetrical with respect to *η*. More generally they should be replaced by periodic boundary conditions on $S=\pm \frac{1}{2}$.
— Equation (4.25) should read
  4.25' $$\begin{array}{rl} \Phi (N,S,s) & =-\frac{\partial \phi_{-1}^{-}}{\partial n}(s)\Phi^{-}(N,S)\\ & \;-\varepsilon \kappa (s)\frac{\partial \phi_{-1}^{-}}{\partial n}(s){\tilde{\Phi }}^{-}(N,S)\\ & \;-\varepsilon \frac{\partial^{2}\phi_{-1}^{-}}{\partial n\partial s}(s){\hat{\Phi }}^{-}(N,S)\\ & \;+\varepsilon \frac{\partial \phi_{0}^{+}}{\partial n}(s)\Phi^{+}(N,S)\\ & \;-\varepsilon \frac{\partial \phi_{0}^{-}}{\partial n}(s)\Phi^{-}(N,S)+O(\varepsilon^{2}) \end{array}$$
— Equations (4.26) and (4.27) should read
  4.26' $$\begin{array}{rl} & -(\tau_{-}-\varepsilon \kappa {\tilde{\tau }}_{-})\frac{\partial \phi_{-1}^{-}}{\partial n}-\varepsilon {\hat{\tau }}_{-}\frac{\partial^{2}\phi_{-1}^{-}}{\partial n\partial s}\\ & \;+\varepsilon \sigma_{+}\frac{\partial \phi_{0}^{+}}{\partial n}-\varepsilon \tau_{-}\frac{\partial \phi_{0}^{-}}{\partial n}\\ & \;=\phi_{0}^{+}+\varepsilon \phi_{1}^{+}\;\text{on }\Gamma \end{array}$$
  and
  4.27' $$\begin{array}{rl} & -(\sigma_{-}-\varepsilon \kappa {\tilde{\sigma }}_{-})\frac{\partial \phi_{-1}^{-}}{\partial n}-\varepsilon {\hat{\sigma }}_{-}\frac{\partial^{2}\phi_{-1}^{-}}{\partial n\partial s}\\ & +\varepsilon \tau_{+}\frac{\partial \phi_{0}^{+}}{\partial n}-\varepsilon \sigma_{-}\frac{\partial \phi_{0}^{-}}{\partial n}\\ & =\frac{1}{\varepsilon }\phi_{-1}^{-}+\phi_{0}^{-}+\varepsilon \phi_{1}^{-}\;\mathrm{on}\;\Gamma \end{array}$$
— The two lines following equation (4.30) should read:
  where $(\nabla^{2}+k_{*}^{2}){\hat{\phi }}_{0}^{+}=f$ in *Ω*_+_ with ${\hat{\phi }}_{0}^{+}=0$ on *Γ*, and $(\nabla^{2}+k_{*}^{2}){\tilde{\phi }}_{0}^{+}=0$ in *Ω*_+_ with ${\tilde{\phi }}_{0}^{+}=-\partial \psi /\partial n$ on *Γ*, with both ${\hat{\phi }}_{0}^{+}$ and ${\tilde{\phi }}_{0}^{+}$ satisfying the Sommerfeld radiation condition at infinity.
— The line following equation (4.33) should read:
  where ${\tilde{\phi }}_{0}^{-}$ is a particular solution of $(\nabla^{2}+k_{*}^{2}){\tilde{\phi }}_{0}^{-}=(I_{2}/I_{1})\psi$ in *Ω*_−_ with ${\tilde{\phi }}_{0}^{-}=-\partial \psi /\partial n$ on *Γ*, and
— Equation (A 18) should read:
  A 18' $$\begin{array}{rl} \Phi & ∼\mp \frac{1}{2}\varepsilon \kappa A_{0}^{\pm }N^{2}\pm (A_{0}^{\pm }+\varepsilon A_{1}^{\pm })N+A_{0}^{\pm }\sigma_{\pm }+A_{0}^{\mp }\tau_{\mp }\\ & \;+\varepsilon A_{1}^{\pm }\sigma_{\pm }+\varepsilon A_{1}^{\mp }\tau_{\mp }\pm \varepsilon \kappa A_{0}^{\pm }{\tilde{\sigma }}_{\pm }\mp \varepsilon \kappa A_{0}^{\mp }{\tilde{\tau }}_{\mp }+\varepsilon \frac{\partial A_{0}^{\pm }}{\partial s}{\hat{\sigma }}_{\pm }+\varepsilon \frac{\partial A_{0}^{\mp }}{\partial s}{\hat{\tau }}_{\mp }+O(\varepsilon^{2}) \end{array}$$

